# Increased levels of antibodies to synaptopodin and annexin 1 in patients with primary podocytopathies

**DOI:** 10.3389/fneph.2024.1471078

**Published:** 2024-10-31

**Authors:** Natalia V. Chebotareva, Evgeniya A. Charionovskaya, Evgenia A. Biryukova, Anatoliy A. Vinogradov, Igor I. Alentov, Natalia S. Sergeeva, Alexey S. Kononikhin, Evgeny N. Nikolaev, Sergey V. Moiseev

**Affiliations:** ^1^ Tareev Clinic of Internal Diseases, Sechenov First Moscow State Medical University, Moscow, Russia; ^2^ Faculty of Medicine, Lomonosov Moscow State University, Moscow, Russia; ^3^ Department of Prediction of Conservative Treatment Efficiency, Hertsen Moscow Oncology Research Institute, Moscow, Russia; ^4^ Project Center of Advanced Mass, Spectrometry Technologies, Skolkovo Institute of Science and Technology, Moscow, Russia

**Keywords:** minimal change disease, focal segmental glomerulosclerosis, podocytopathy, annexin 1, synaptopodin

## Abstract

**Introduction:**

Circulating anti-podocyte antibodies have been proposed as potential factors contributing to increased permeability in primary podocytopathies, such as Minimal Change Disease (MCD) and Focal Segmental Glomerulosclerosis (FSGS). The aim of the study was to to assess the levels of antibodies targeting synaptopodin and annexin 1 in the blood serum of patients diagnosed with nephrotic syndrome, with the aim of evaluating their potential utility in diagnosing primary podocytopathies and predicting therapeutic response.

**Methods:**

The study included a total of 72 patients diagnosed with nephrotic syndrome, alongside 21 healthy subjects for comparison. Among the patients, 38 were diagnosed with FSGS, 12 with MCD, and 22 with MN. The levels of anti-synaptopodin and anti-annexin-1 antibodies were quantified using Enzyme-Linked Immunosorbent Assay.

**Results:**

The levels of antibodies to annexin 1 and anti-synaptopodin in the blood were found to be higher in patients diagnosed with MCD and FSGS compared to those with MN and healthy individuals. The elevated levels of antibodies to annexin 1 and synaptopodin showed area under the curve values of 0.826 (95% CI 0.732–0.923) and 0.827 (95% CI 0.741–0.879), respectively. However, a model incorporating both antibodies demonstrated higher sensitivity (80.9%) and specificity (81.3%) with an AUC of 0.859 (95% CI 0.760-0.957). Notably, serum levels of annexin 1 and anti-synaptopodin antibodies did not predict the response to prednisolone and/or CNI therapy.

**Discussion:**

Levels of antibodies targeting synaptopodin and annexin 1 were notably elevated in patients diagnosed with MCD and FSGS compared to those with MN and healthy controls. A panel comprising both antibodies demonstrated moderate to high sensitivity and specificity for diagnosis MCD or FSGS.

## 1 Introduction

The pathogenesis of primary podocytopathies, including MCD and FSGS, remains poorly understood. The notion of a circulating permeability factor has been extensively discussed in recent literature (1, 2). Evidence supporting the existence of these factors primarily derives from *in vitro* and animal studies (3–5).

However, identifying a singular factor specific to human MCD/FSGS remains elusive. Immune response dysregulation, involving immune cells and soluble factors, is a consistent finding in patients with podocytopathies. (6, 7). Notably, an imbalance of Th17 and Regulatory cells has been established in patients with MCD and FSGS (8–10). Moreover, the efficacy of B-cell depletion therapy underscores the significant involvement of the B-cell component in the pathogenesis of these diseases (11–13). Published in 2022, a study on the discovery of autoantibodies targeting nephrin in MCD found them in 29% of patients, further strengthening this hypothesis (14). Prior to the discovery of nephrin antibodies, some studies had already shown increased levels of various antibodies in MCD. These included anti-UCHL1 antibodies, anti-annexin 2 antibodies, and anti-CD40 antibodies in idiopathic NS among children, as well as in cases of FSGS relapse in kidney transplant recipients. (15–17).

Less is known about the production of these antibodies in adults. In our previous studies involving a small cohort of patients with various glomerulopathies, we observed varying degrees of antibody elevation, including antibodies to nephrin, anti-UCHL1 antibodies, and anti-CD40 antibodies in the MCD and FSGS groups. However, the sensitivity and specificity of these antibodies do not currently support their consideration as diagnostic tests (18, 19).

Interest in factors contributing to structural and functional damage to podocytes is driven by well-described processes such as podocyte foot effacement and detachment from the GBM, which underlie nephrotic syndrome (20, 21). Furthermore, irreversible damage leading to critical podocyte loss appears to influence prognosis and disease outcome (22). The contribution of anti-podocyte antibodies, while detected in these patients, remains unclear in primary podocytopathies. In our present work, we examined the levels of antibodies targeting other structural and functional components of podocytes—synaptopodin, a cytoskeletal component of podocytes, and annexin 1, a surface molecule on podocytes—in a cohort of patients with previously assessed levels of anti-nephrin antibodies.

## 2 Materials and methods

### 2.1 Study cohort

The study comprised 72 patients diagnosed with podocytopathies, including 37 women (51.4%) and 35 men (48.6%), with ages ranging from 18 to 75 years and a median age of 43 years (interquartile range: 27.8-57.5 years). Additionally, a control group (n=21) was established, consisting of 11 women (52.4%) and 10 men (47.6%), aged between 20 and 50 years, with a median age of 32 years (interquartile range: 21-46 years).

The study received ethical approval from the Ethics Committee of Sechenov University (Protocol 30-20, 21st October 2020) and adhered to the principles outlined in the Declaration of Helsinki. Informed consent was obtained from all individual participants included in the study.

### 2.2 Determination of anti-annexin1 and anti-synaptopodin antibodies in serum

To detect antibodies, serum was obtained by centrifuging whole blood at room temperature for 20 minutes at 3,000 rpm. Serum was drawn before the start of therapy. Antibodies targeting annexin 1 (AEE787Hu, Cloud-Clone Corp., BlueGene, Elabscience Biotechnology, USA) and synaptopodin (AEС885Hu, Cloud-Clone Corp., BlueGene, Elabscience Biotechnology, USA) were analyzed using an enzyme immunoassay. According to the manufacturer’s instructions, the detection range for these antibodies is 3.12-200 ng/ml. In each well, 100 μL of diluted standard, blank, and sample solutions were added and incubated for 1 hour at 37°C. Subsequently, 100 μL of the Detection Reagent working solution was added to each well and incubated for an additional hour at 37°C. Following this, 90 μL of Substrate Solution was added to each well, followed by a 20-minute incubation period. The final step involved adding 50 μL of Stop Solution. Immediate measurements were taken at 450 nm using a microplate reader.

According manufacture instruction Intra-assay precision (Precision within an assay): CV<10%, Inter-assay precision (Precision between assays): CV<12% for anti-synaptopodin and anti-annexin 1 antibodies.

### 2.3 Statistical analysis

The data analysis utilized techniques from both the Medcalc Version 22.009 and Jamovi software packages, employing methods of variance statistics. Clinical and laboratory data were described using the median and interquartile range (IQR), representing the 25th and 75th percentiles. Nonparametric tests were applied for both pairwise and multiple comparisons. For comparisons between two independent groups, the Mann-Whitney test was utilized. When dealing with three or more independent groups, a Kruskal-Wallis ANOVA was conducted, with a Dwass-Steel-Crichlow-Fligner correction employed for pairwise comparisons. To evaluate and identify relationships between the studied indicators, Spearman’s non-parametric method of rank correlation was used. Statistical significance was determined at a threshold of p<0.05.

## 3 Results

### 3.1 Characteristics of the patients

The study included 72 patients with podocytopathies. Upon histological examination, 38 patients were diagnosed with FSGS, while 12 were diagnosed with MCD. Additionally, 22 patients tested positive for anti-phospholipase-A2 receptor (PLA2R) autoantibodies, representing a comparison group with membranous nephropathy. Notably, the study included cases of primary FSGS during active disease, meeting NS criteria, including proteinuria exceeding 3.5 g/day, hypoalbuminemia below 30 g/l, and hyperlipidemia. Genetic FSGS was ruled out through whole genome sequencing in patients below 30 years of age who presented with steroid-resistant NS.

The extent of tubulointerstitial fibrosis was assessed using a semi-quantitative scoring system: a score of 1 indicated fibrosis involving less than 25% of the tissue, a score of 2 indicated fibrosis spanning 25-50% of the tissue, and a score of 3 indicated substantial interstitial fibrosis beyond 50%. Additionally, the proportion of sclerosed glomeruli was determined as a percentage relative to the total glomerular count within the biopsy sample.

Twenty-four patients diagnosed with FSGS and eight patients with MCD were closely monitored to assess the impact of immunosuppressive therapy, with corticosteroids (n=28) or cyclosporine (n=6) chosen as the initial approach. Complete remission was defined as a reduction in proteinuria to below 300 mg/day while maintaining stable renal function. Patients who did not respond adequately to corticosteroids for over 16 weeks, with either satisfactory tolerance for more than 8 weeks or poor tolerance for more than 8 weeks, or to cyclosporine for a period of 6 months, were considered non-responders.

The characteristics of the patients examined are presented in **Table 1**.

**Table 1:** Characteristics of the patients.

Parameters	FSGS n=38	MCD n=12	MN n=22
Age, years	38.5 [27-58.2]	39 [28-55,2]	51 [40.5-66.8]
Males, n (%)	17 (44.7)	2 (17)	16 (73)
Proteinuria, g/24h	5.2 [4.09-9.4]	4.37 [2.97-8.00]	5.98 [3.43-7.57]
Serum albumin, g/l	20.8 [17.05-25.1]	21.7 [20.2-31]	25.5 [20.75-29.2]
Duration of nephrotic syndrome, months	17 [5.75-45.75]	1 [1-7,5]	5 [2.25-15]
Serum creatinine, μmol/l	113.5 [78.2-175,6]	90.45 [76.25-161.8]	108.0 [92.45-139.3]
eGFR, ml/min/1,73 m^2^	59 [37.5-89]	78.5 [33-96]	68 [54.8-87.5]
Arterial hypertension, n (%)	28 (73.6)	0	18 (82)
Glomerulosclerosis	0 [0-5]	0	0 [0-2.5]
Tubulo-interstitial fibrosis >25%	9 (23.7)	0	8(36.3)
Corticosteroid/cyclosporine-resistant NS, n (%)	13 out of 24 (54.2)	0	–
Corticosteroid/cyclosporine-sensitive NS, n (%)	11 out of 24 (45.8)	8 out of 8 (100)	–

The table shows the median [25 - 75 %].

### 3.2 Anti-annexin 1 antibodies in serum

**Figure 1:**
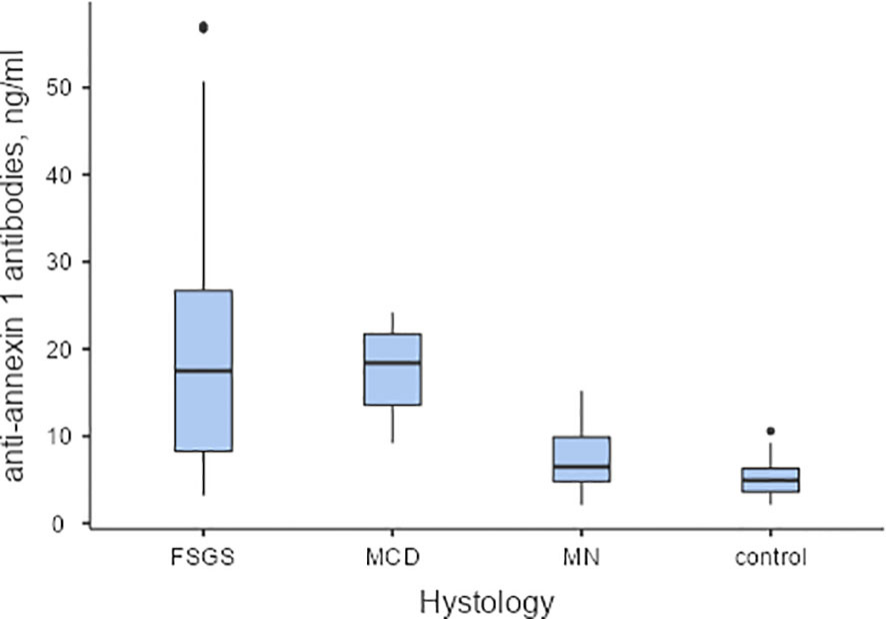
The baseline levels of anti-annexin 1 antibodies in serum of patients with podocytopathies, and the control group. Pairwise Dwass-Steel-Critchlow-Flinger comparison: MCD vs. FSGS W-0.708, p=0.959; MCD vs.MN W-4.831, p=0.004; MCD vs. control W-5.437, p<0.001; FSGS vs. MN W-4.795, p=0.004; FSGS vs. control W-5.764, p<0.001; MN vs. control W-2.657, p=0.237. MCD – minimal change disease; FSGS, focal segmental glomerulosclerosis; MN, membranous nephropathy.

The level of antibodies to annexin 1 in the blood was elevated in patients diagnosed with MCD and FSGS compared to those with MN and healthy individuals. (Refer to **Figure 1**)

**Figure 2:**
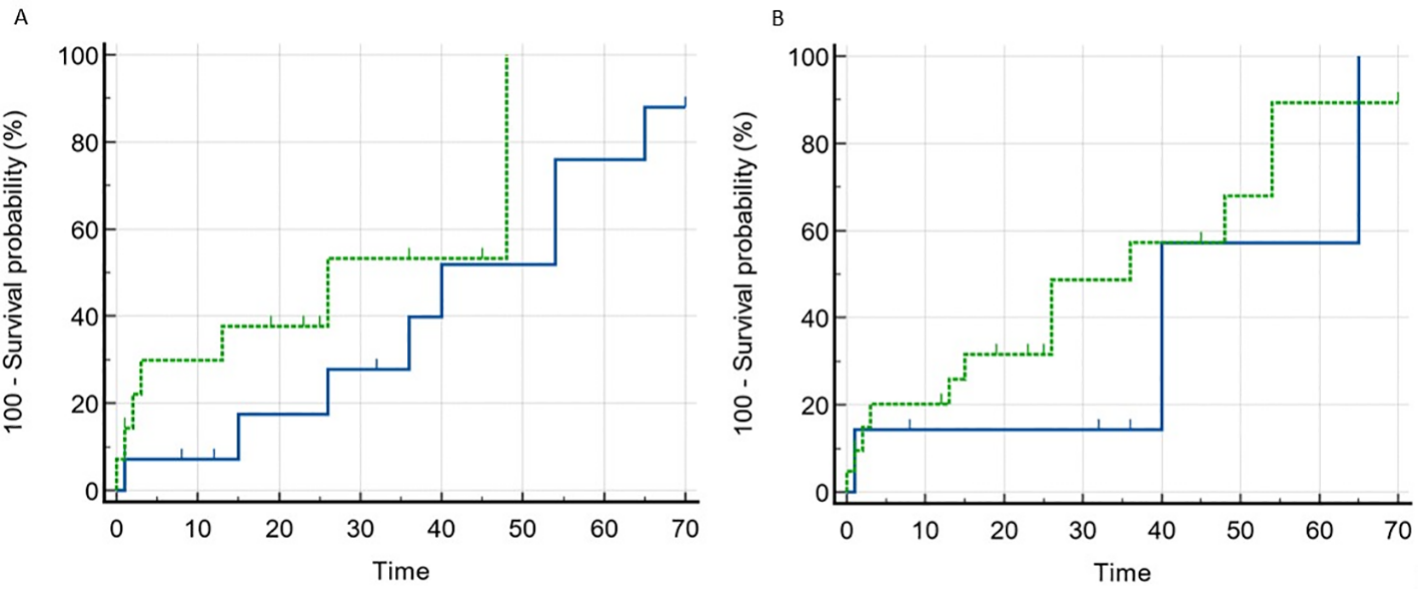
Remission rate depending on the baseline level of anti-annexin 1 and anti-synaptopodin antibodies in patients with MCD and FSGS. **(A)**. Blue line – anti-annexin 1 antibody negative, dotted line – anti-annexin 1 antibody positive. Comparison of curves (Log-rank test): Chi-squared 1.698, p=0.193. **(B)**. Blue line – anti-synaptopodin antibody negative, dotted line – anti-synaptopodin antibody positive. Comparison of curves (Log-rank test): Chi-squared 0.556, p=0.456.

In a unifactorial logistic regression model, the level of antibodies to annexin 1 was found to be influenced by the degree of serum albumin (OR 0.901, 95% CI 0.814-0.998, p=0.045) and the interstitial fibrosis score (OR 0.12, 95% CI 0.028-0.521, p=0.005). Anti-annexin 1 antibodies in the blood were observed to be higher in patients with more severe nephrotic syndrome and in the absence of tubulointerstitial fibrosis. However, serum annexin 1 antibody levels did not predict response to prednisolone and/or calcineurin inhibitors therapy (Refer to **Figure 2A**).

### 3.3 Anti-synaptopodin antibodies in serum

**Figure 3:**
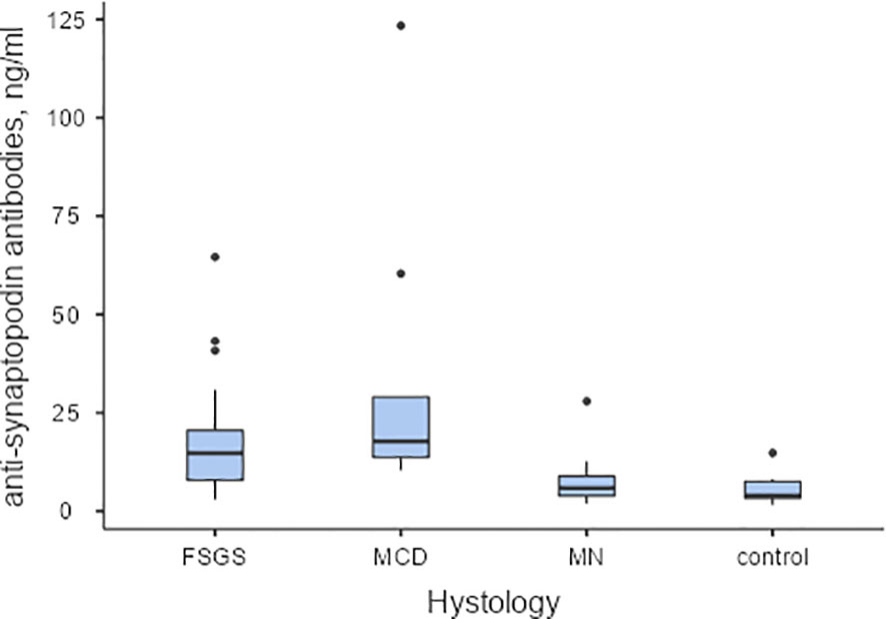
The baseline levels of anti-synaptopodin antibodies in serum of patients with podocytopathies, and the control group Pairwise Dwass-Steel-Critchlow-Flinger comparison: MCD vs. FSGS W-1.26, p=0.809; MCD vs. MN W-5.32, p<0.001; MCD vs. control W-5.36, p<0.001; FSGS vs. MN W-4.60, p=0.006; FSGS vs. control W-5.46, p<0.001; MN vs. control W-2.35, p=0.346.

Serum levels of antibodies to synaptopodin were found to be higher in patients diagnosed with MCD and FSGS compared to those with MN and healthy controls (Refer to **Figure 3**). Additionally, antibodies to synaptopodin were lower in patients over 45 years of age (OR 0.943, 95% CI 0.907-0.981, p=0.004) and in the presence of tubulointerstitial fibrosis greater than 25% (OR 0.182, 95% CI 0.047-0.709, p=0.014). However, the level of antibodies to synaptopodin in the blood did not affect the response to immunosuppressive therapy with prednisolone and/or calcineurin inhibitors (Refer to **Figure 2B**). When considering the upper limit of the reference interval for subjects without kidney disease, it is noteworthy that all patients with MCD tested positive for both anti-annexin 1 and anti-synaptopodin antibodies.

### 3.4 Level of antibodies to annexin1 and synaptopodin for the diagnosis of podocytopathies

In the FSGS group, an increase in the level of both antibodies to annexin 1 and synaptopodin was observed in 20 (52.6%) out of 38 patients, while an increased level of one of the two antibodies was noted in 10 (26.3%) patients, and no increase in antibodies was observed in 8 (21.1%) patients. Among patients with membranous nephropathy, both antibody levels were elevated in one patient, one of the antibody levels was elevated in 5 patients, and the majority (16 out of 22, or 72.7%) of patients did not differ from the healthy control group in antibody levels.

Since the studied antibodies to annexin 1 and synaptopodin (either alone or in combination) were elevated in the majority of patients with MCD and FSGS, we sought to compare the diagnostic accuracy of these tests for the diagnosis of primary podocytopathies vs. membranous nephropathy. Cut-off for anti-annexin 1 antibodies is 15.18 ng/ml; sensitivity – 56.2%, specificity 95.2%; PPV – 94.7%; NPV – 58.8%; Youden’s index – 0.515; AUC – 0.826 (95% CI 0.732–0.923). Cut-off for anti-synaptopodin antibodies is 10.2 ng/ml; sensitivity – 78.12%, specificity 90.48%; PPV – 92.6%; NPV – 73.1%; Youden’s index – 0.686; AUC 0.827 (95% CI 0.741–0.879). Elevated antibodies to annexin 1 and synaptopodin exhibited high specificity (95.2%, and 90.5%, respectively) but low sensitivity (56.3%, and 78.12%, respectively). However, considering the increase in both antibodies simultaneously, the sensitivity of the model increased to sensitivity – 81.25%, specificity 81.00%; PPV – 86.67%; NPV – 73.91%; Youden’s index – 0.622; resulting in an AUC of 0.862 (95% CI 0.764-0.959).

## 4 Discussion

In the present study, we evaluated the levels of antibodies to synaptopodin, a proline-rich protein associated with actin microfilaments found in the foot processes of podocytes. Previous research has suggested that higher synaptopodin expression in podocytes correlates with a significantly better response to steroid therapy (23). Additionally, a study by Wang RX et al. indicated the expression of annexin 1 in podocytes (24). In our study, we excluded patients with mutations in genes encoding podocyte proteins and collagen, as well as those with FSGS without nephrotic syndrome and possible secondary forms of FSGS. This allowed us to identify a cohort of patients with primary idiopathic FSGS. We observed a significant increase in the levels of antibodies to both synaptopodin and annexin 1 in adult patients with MCD and FSGS presenting with nephrotic syndrome, compared to those with membranous nephropathy and healthy individuals. However, our results also revealed elevated levels of antibodies in patients with MN. Furthermore, we found no association between the levels of antibodies to synaptopodin and annexin 1 and the response to therapy with corticosteroids and calcineurin inhibitors. The presence of annexin1 and synaptopodin antibodies detected by ELISA seems not to predict clinical response and course of the disease in patients with primary podocytopathies and its pathogenic role remains not clear.

The significant increase in antibody levels observed in patients with MCD and some patients with FSGS leads us to hypothesize antibody-mediated damage to podocytes and potential B-cell dysregulation (11). However, a counterargument arises from previous published work, similar to the findings regarding antibodies to nephrin. Specifically, the levels of antibodies to other components of podocytes, such as synaptopodin and annexin 1, were not associated with the response to immunosuppressive therapy in patients with podocytopathies (14).

Our findings also revealed a noteworthy trend: levels of synaptopodin antibodies tend to decrease with age, suggesting a potentially less active immune response in adults. Moreover, in patients with more severe tubulointerstitial fibrosis, the levels of both antibodies were lower, implying a potential decrease as the disease progresses, particularly at later stages marked by the development of fibrosis in the kidney. Unlike animal models where disease onset can be precisely established, such as (25–27), in humans, pinpointing the exact onset of the disease is challenging. Consequently, we cannot entirely rule out the pathological role of anti-podocyte antibodies in the initial damage to podocytes, nor can we disregard the possibility of a decline in antibody levels over time. Severe podocyte damage at disease onset and the subsequent development of podocytopenia may serve as a trigger for epithelial-mesenchymal transformation and fibrosis processes within the kidney (28).

It is intriguing that in podocytopathies, particularly in MCD, the levels of antibodies to various podocyte antigens are elevated. However, the determination of individual antibodies alone does not suffice as a sensitive diagnostic test for identifying primary podocytopathies (MCD and FSGS comparing membranous nephropathy), although the diagnostic accuracy of antibodies to synaptopodin appears to be better. For comparison, aPLA2R exhibits a sensitivity of 65% (63–67%), specificity of 97% (97–98%), and an AUC of 0.939 for diagnosis of MN (29). Nonetheless, by evaluating the increase in both antibodies to synaptopodin and annexin 1, we can enhance sensitivity to 85.7% and specificity of 81.2%, with an AUC of 0.862 (95% confidence interval [CI] 0.764-0.959) for the diagnosis of podocytopathies (MCD and FSGS comparing membranous nephropathy). Since in the case of combination a non-specific increase in both antibodies to synaptopodin and annexin 1 is possible, the specificity decreases. However, if the optimal balance of sensitivity and specificity is considered, then combination of two antibodies is preferable. This suggests the potential utility of developing a panel, possibly incorporating other anti-podocyte antibodies, such as those targeting nephrin, to further enhance the diagnostic accuracy of tests for primary podocytopathies. However, this hypothesis requires further validation in larger cohorts.

Similar to antibodies targeting nephrin, the role of antibodies against synaptopodin and annexin 1 in predicting response to immunosuppressive therapy remains unclear. This uncertainty may stem from variations in the duration of the disease prior to antibody testing, as well as differences in the severity of podocytopenia and fibrosis among patients. Moreover, the mechanism by which immune cells are activated to produce various antibodies targeting different molecular structures on the surface of podocytes, recognized by the immune system, remains incompletely understood (11). The discovery of numerous anti-podocyte antibodies in primary podocytopathies by various research groups raises questions about whether these antibodies are causative agents or mere indicators of damage. The presence of anti-annexin1 and synaptopodin antibodies detected by ELISA seems not to predict clinical course of the disease in patients with primary podocytopathies and its pathogenic role remains not clear. It should be noted that the same trend is characteristic of other antibodies, for example, antibodies to deoxyribonucleic Acid (DNA) in systemic lupus erythematosus or antineutrophil cytoplasmic antibody (ANCA) in ANCA-associated vasculitis, which can remain elevated even in remission. For example, despite a good association between anti-DNA autoantibodies and lupus nephritis, it is difficult to determine the pathogenic potential of an anti-DNA autoantibody response. Reactivity to double stranded DNA is considered as one of the characteristic of polyreactive autoantibodies and not a primary requisite for the pathogenesis of lupus nephritis (30). Various hypotheses with different kinds of triggers have been suggested concerning ANCA formation, none has been confirmed to date. Lower titre, lower avidity, and lack of IgG3 subclass of these autoantibodies associated with their non-pathogenic co-existence in serum, but whether ANCA are targeting these epitopes remaining non-pathogenic at higher concentrations, remains unclear (31, 32).

It is possible that the presence of these annexin-1 and synaptopodin antibodies is just an indirect marker of podocyte injury, and not the causal factor or the primary mechanism of podocyte`s damage, since many serum samples showed an increase in antibodies to synaptopodin and annexin 1. Considering the release of numerous antigens/epitopes, both surface and intracellular, during podocyte damage—such as the actin cytoskeleton and slit diaphragm—against which a secondary immune response can develop, antibodies may serve as indicators of podocyte damage. However, the trigger factor for such immune activation remains elusive and requires further research. In particular, the pathogenic role of antibodies can be proven from morphological studies demonstrating binding of antibodies to podocyte antigens or in animal experiments or ex vivo in cell culture (33).

One limitation of our study is the small number of patients within each group, not homogenous case-control groups which may limit the generalizability of our findings. Since the results were obtained from a relatively small groups of patients, a multicenter study is needed in order to confirm the data on anti-aannexin 1 and anti-synaptopodin antibodies in podocytopathies. Additionally, the absence of antibody dynamics over the course of immunosuppressive therapy and variations in the duration of NS prior to antibody assessment are also notable limitations.

In conclusion, our study found significantly higher levels of antibodies to synaptopodin and annexin 1 in patients with podocytopathies, specifically MCD and FSGS, compared to those with MN and healthy individuals. Elevated levels of both anti-annexin 1 and anti-synaptopodin antibodies concurrently exhibited moderate to high sensitivity and specificity for the diagnosis of MCD or FSGS. However, these elevated antibody levels were not associated with the likelihood of achieving remission.

## Data Availability

The raw data supporting the conclusions of this article will be made available by the authors, without undue reservation.
